# Frequency of Th17 cells correlates with the presence of lung lesions in pigs chronically infected with *Actinobacillus pleuropneumoniae*

**DOI:** 10.1186/s13567-017-0411-z

**Published:** 2017-02-06

**Authors:** Elena L. Sassu, Andrea Ladinig, Stephanie C. Talker, Maria Stadler, Christian Knecht, Heiko Stein, Janna Frömbling, Barbara Richter, Joachim Spergser, Monika Ehling-Schulz, Robert Graage, Isabel Hennig-Pauka, Wilhelm Gerner

**Affiliations:** 1 University Clinic for Swine, Department of Farm Animals and Veterinary Public Health, University of Veterinary Medicine, Vienna, Austria; 2 Institute of Immunology, Department of Pathobiology, University of Veterinary Medicine Vienna, Vienna, Austria; 3 Functional Microbiology, Institute of Microbiology, Department of Pathobiology, University of Veterinary Medicine Vienna, Vienna, Austria; 4 Institute of Pathology and Forensic Veterinary Medicine, Department of Pathobiology, University of Veterinary Medicine Vienna, Vienna, Austria; 5 Division of Swine Medicine, Department of Farm Animals, University of Zurich, Vetsuisse Faculty, Zurich, Switzerland

## Abstract

Porcine contagious pleuropneumonia caused by *Actinobacillus pleuropneumoniae* (APP) remains one of the major causes of poor growth performance and respiratory disease in pig herds. While the role of antibodies against APP has been intensely studied, the porcine T cell response remains poorly characterized. To address this, pigs were intranasally infected with APP serotype 2 and euthanized during the acute phase [6–10 days post-infection (dpi)] or the chronic phase of APP infection (27–31 dpi). Lymphocytes isolated from blood, tonsils, lung tissue and tracheobronchial lymph nodes were analyzed by intracellular cytokine staining (ICS) for IL-17A, IL-10 and TNF-α production after in vitro stimulation with crude capsular extract (CCE) of the APP inoculation strain. This was combined with cell surface staining for the expression of CD4, CD8α and TCR-γδ. Clinical records, microbiological investigations and pathological findings confirmed the induction of a subclinical APP infection. ICS-assays revealed the presence of APP-CCE specific CD4^+^CD8α^dim^ IL-17A-producing T cells in blood and lung tissue in most infected animals during the acute and chronic phase of infection and a minor fraction of these cells co-produced TNF-α. APP-CCE specific IL-17A-producing γδ T cells could not be found and APP-CCE specific IL-10-producing CD4^+^ T cells were present in various organs but only in a few infected animals. The frequency of identified putative Th17 cells (CD4^+^CD8α^dim^IL-17A^+^) in lung and blood correlated positively with lung lesion scores and APP-specific antibody titers during the chronic phase. These results suggest a potential role of Th17 cells in the immune pathogenesis of APP infection.

**Electronic supplementary material:**

The online version of this article (doi:10.1186/s13567-017-0411-z) contains supplementary material, which is available to authorized users.

## Introduction

*Actinobacillus pleuropneumoniae* (APP) is a gram negative bacterium, belonging to the *Pasteurellaceae* family that causes porcine respiratory disease worldwide. The outcome of the infection can vary from sudden death with bloody nasal discharge to an acute disease with fever and coughing that frequently results in chronic infections [1]. Vaccination and antibiotic based therapies can help to reduce the severity of the symptoms and decrease the mortality rates, but are not effective in clearing the bacteria [2]. In fact, pigs overcoming the acute phase can become subclinically infected and persistent carriers, harboring APP in tonsils and chronic lung lesions [3]. Since 1957, when APP was first reported, most research activities were focused on the elucidation of the humoral immune response [4–6]. Thereby it also became clear that APP developed several strategies to avoid humoral host defense mechanisms. For example, in vitro experiments indicated that APP can survive in alveolar macrophages [7], has the capacity for enhanced biofilm formation in anaerobic conditions [8], and changes the polysaccharide composition of the capsule [3]; all possibly contributing to an escape from humoral immunity and to the establishment of chronic infection in lung tissue and tonsils.

For a more thorough understanding of APP pathogenesis and persistence, cell-mediated immune mechanisms also need to be taken into focus. In particular, T-cell responses may equip the host with additional means to combat APP infections, but could also be involved in dysfunctional immune responses [9] or could support immune escape mechanisms [10]. Hitherto, the T-cell mediated immune response to APP has been poorly characterized in swine. Early studies indicated the potential relevance of T cells, because the intensity of a T-cell dependent delayed-type hypersensitivity reaction was associated with protection against an APP challenge infection [11]. In addition, a change in the CD4:CD8 ratio in peripheral blood following low-dose APP immunization and high-dose APP challenge has been reported, but the phenotype of involved cells was not further studied [12]. Furthermore, Faldyna et al. [13] described an increase of CD8α^−^ γδ T cells in bronchoalveolar lavage fluid (BALF) as well as B-cells in tracheobronchial lymph nodes of pigs challenged with APP suggesting a role of γδ T cells in this infection. More recently, IL-17 was shown to be induced on the transcriptional level in lungs of pigs affected by APP [14] and it has been demonstrated that CD4^+^ and γδ T cells are capable to produce IL-17 in swine [15–17]. From studies in mice and humans it is known that IL-17-producing CD4^+^ T (Th17) cells are involved in the clearance of extracellular pathogens in peripheral organs by attraction and stimulation of neutrophils [18]. There is also some evidence that Th17 cells can be involved in chronic airway inflammation [19]. Moreover, in vivo and in vitro studies with *Mannheimia haemolytica*, which like APP belongs to the *Pasteurellaceae* family and induces neutrophilic infiltration in the lung, suggested an IL-17 production by bovine γδ T cells [20]. Thus we hypothesized that IL-17 production by Th17 but also γδ T cells might be involved in the porcine immune response to APP. Since the anti-inflammatory cytokine IL-10 may support the survival of microorganisms in the host via inhibiting their cell-mediated immune response [21–23], we investigated in parallel its role in persistence of APP.

To address these issues we developed an APP infection model and an in vitro stimulation assay making use of an APP crude capsular extract (APP-CCE). Cytokine production by CD4^+^ and γδ T cells was investigated by intracellular cytokine staining (ICS) of lymphocytes isolated from different host compartments during the acute and chronic phase of APP infection. We found that the majority of pigs infected with APP harbor APP-CCE specific IL-17A^+^ CD4^+^ T cells in the lung and in the blood during the acute and the chronic phase of APP infection. In chronically infected animals, the frequency of these cells in lung and peripheral blood was found to correlate positively with lung lesions and APP-specific antibody titers.

## Materials and methods

### Experimental APP infection model

Thirty 5-week-old male castrated pigs (German Landrace), routinely tested to be negative for APP, porcine reproductive and respiratory syndrome virus (PRRSV), toxigenic *Pasteurella multocida*, endo- and ectoparasites, were derived from a closed breeding herd of high health status in Mecklenburg-Western Pomerania, Germany. Animals were moved to Austria, following European guidelines on protecting the welfare of animals during transport, stated by Regulation (EC) No 1/2005. Upon arrival, animals entered a biosafety level 2 facility at the University of Veterinary Medicine Vienna, where they were kept for the entire duration of the experiment. Animals were weighed, individually marked with ear tags, and then, according to their body weight, divided into a control and an infected group of ten and twenty animals respectively. Control and infected group were housed in separate compartments. Within the infected group, animals were assigned to two subgroups of ten animals each, which were kept under identical conditions but euthanized either 6–10 days post-infection (dpi) (acute infected group) or 27–31 dpi (chronic infected group). At the time of arrival, the APP-free status of the pigs was confirmed by bacteriological examination of nasal and tonsillar swabs and by serological testing for antibodies against Apx-IV using the commercially available IDEXX APP-ApxIV Ab Test ELISA (IDEXX Laboratories, Westbrook, USA). After 2 weeks of adaptation, at day 0 an intranasal spray infection was performed. For the infection, an APP biotype 1 serotype 2 strain (Lab number C3656/0271/11) was used, isolated originally by the Institute of Microbiology, University of Veterinary Medicine, Hanover, Germany from a diseased fattening pig during an acute outbreak of porcine pleuropneumonia in northern Germany [24]. After initial isolation, bacteria were animal-passaged once and lab-passaged four times in PPLO medium supplemented with NAD. Pigs were infected with 2 mL (1 mL into each nostril) of bacterial culture containing 2 × 10^4^ CFU/mL. The bacterial culture was vaporized directly into the nostrils of the pigs by using a mucosal atomization device (LMA MAD Nasal™, Teleflex Medical GmbH, Athlone, Ireland). Control pigs underwent the same procedure, but received 2 mL of 154 mM sterile NaCl instead of the bacterial culture. Daily clinical examinations were carried out and assembled in a clinical score, considering rectal temperature, presence of dyspnea and/or coughing and changes in behavior (see Additional file 1 for details). Additionally, pig body weights were recorded weekly. To screen for presence of APP in the upper respiratory tract, nasal and tonsillar swabs were examined at 14 and 21 dpi. At the end of the experiment, after animals were euthanized, tonsillar tissues were taken instead of swabs. Blood samples were taken by puncture of the *V. cava cranialis* or *V. jugularis* on the same days. Sera were used for detection of APP 2 specific antibodies, while heparinized samples were obtained to isolate peripheral blood mononuclear cells (PBMCs). Euthanasia was performed on five consecutive days (two infected pigs and one control pig per day) in two different time frames: 6–10 dpi and 27–31 dpi for the acutely and the chronically infected group, respectively. Within these two periods, animals were randomly selected for euthanasia, which was performed by intracardial administration of T61^®^ (T61^®^: Embutramid, Mebezoniumiodid, Tetracainhydrochlorid, 1 mL/10 kg BW, MSD, Whitehouse Station, NJ, USA) during anesthesia (Narketan^®^, Stresnil^®^). All animal procedures were approved by the institutional ethics committee, the Advisory Committee for Animal experiments (§12 of Law for Animal Experiments, Tierversuchsgesetz—TVG) and the Federal Ministry for Science and Research (reference number bmwfw GZ 68.205/0138-WF/V/3b/2015).

### Gross necropsy and pathological examination

At necropsy, a general pathological examination of the carcass was performed, with focus on the respiratory tract. Organs of interest for the study were extracted in the following order: salivary gland (*Glandula mandibularis*, GM), tonsils, tracheobronchial lymph node (TBLN) and lung. After evaluation of the thoracic cavity, the lung was extracted from the chest while paying particular attention to the presence of pleural effusion or pleural adhesions. Then the severity of the pathological findings was determined using the lung lesion score (LLS) by Hannan et al. [25] and using the slaughterhouse pleurisy evaluation system (SPES) [26] for assessment of the pleura status. After clamping off the left main bronchus, the right lung was flushed with 100 mL of 154 mM sterile NaCl for collection of BALF, while tissue samples were taken from the dorsal portion of the left caudal lobe. If no lesions were detected in this particular area, an additional sample from another affected part of the lung was taken for histologic and bacteriological investigations. For histology, samples were fixed in 10% neutral buffered formalin, processed in 3-µm-thick paraffin-embedded sections and stained with haematoxylin and eosin.

### Microbiological investigation

Nasal and tonsillar swabs from living animals and nasal swabs, salivary gland, tonsils, tracheobronchial lymph node, lung and BALF from euthanized animals were investigated for the presence of APP by streaking the samples on Columbia sheep blood agar (Oxoid, Vienna, Austria). *Staphylococcus aureus* was used as nurse to facilitate the isolation of APP from organs carrying a high bacterial background flora, such as tonsils and nose [1]. Subsequently, APP was transferred to PPLO agar supplemented with 10 mg/L NAD (AppliChem GmbH, Darmstadt, Germany). Plates were incubated overnight at 37 °C and 5% CO_2_. Identification of the re-isolated bacteria was confirmed by serotype 2 specific PCR, using primers for the capsular biosynthesis genes *cps2AB* [27]. In addition, snap frozen tissue samples were examined directly by a conventional PCR based on detection of the apxIVA gene [28].

### Determination of APP 2-specific antibody titers in serum

Sera obtained prior to infection (day 0), at the time of euthanasia and at 14 and 21 dpi were analyzed for antibodies against APP 2 using the commercial Swinecheck^®^ APP 2 ELISA (Biovet, St-Hyacinthe, Canada) according to the manufacturer’s instructions. Results were recorded as S/P ratio, obtained by the ratio between optical density (OD) of each sample (S) and the mean OD of the positive control (P): ODs/MODp.

### Preparation of APP crude capsular extract for in vitro recall experiments

To stimulate lymphocytes in vitro, a crude capsular extract (CCE) from the APP serotype 2 strain C3656/0271/11, which has been used to infect the animals, was prepared following a modified protocol from Wittkowski et al. [29]. In detail, 300 mL liquid cultures of APP biotype 1, serotype 2, strain C3656, were grown to an OD_600_ of approximately 0.2 and harvested by centrifugation at 6530 *g* for 5 min. Aqueous phenol (1%, w/v) was added to the harvested bacteria (18 mL per gram of bacterial wet weight). Thereafter, the suspensions were shaken for 10 min at 37 °C and transferred to conical 25 mL flasks and the solution was gently stirred for 4 h at 4 °C. After centrifugation at 21 420 *g* for 30 min at 4 °C, the supernatant was filtrated (0.2 µm, Filtropur, Sarstedt, Nümbrecht, Germany), dialyzed against MilliQ-H_2_O (2–4 L replaced every 4 h during the first day, then every 8 h) at 4 °C for 2 days, using 1 kDa MWCO membrane (Mini Dialysis Kit, GE Healthcare) and finally lyophilized overnight. To preserve the integrity of potential immunogenic proteins in the capsular extract, no further purification was performed. Lyophilized samples were dissolved in phosphate buffered saline (PBS) to reach a final concentration of 1 mg/mL. The stimulus was tested for potential toxicity in ConA-stimulated (3 µg/mL) PBMCs labelled with violet proliferation dye as described elsewhere [30]. After 4 days of cultivation, PBMCs were harvested and stained with Live/Dead^®^ Near-IR stain kit (Invitrogen, Carlsbad, CA, USA) according to manufacturer’s instructions and subjected to flow cytometry (FCM). Frequencies of dead cells and proliferating cells were determined and by a dose titration of the CCE, the optimal working concentration was found to be 4 µg/mL.

### Sample collection and isolation of lymphocytes

Blood samples were collected in Lithium-Heparin tubes (Primavette^®^, KABE Labortechnik, Nümbrecht, Germany) prior to infection (day 0), at 14 and 21 dpi and at the time of death. PBMCs were isolated by density gradient centrifugation (Pancoll human, density 1.077 g/mL, PAN Biotech, Aidenbach, Germany) as described elsewhere [31]. Tonsils and tracheobronchial lymph nodes were subjected to a procedure for isolation of lymphocytes as previously described [30].

For isolation of lymphocytes from lung tissue, a block of tissue (approx. 4 × 3 × 2 cm) from the dorsal portion of the left caudal lobe was cut into small pieces (approx. 3 × 3 × 3 mm) and lymphocytes were isolated as described elsewhere [32]. Cells from the various tissues and blood were counted and suspended in cell culture medium (RPMI1640 with stable glutamine supplemented with 10% FCS, 100 IU/mL penicillin, 100 µg/mL streptomycin, all from PAN Biotech and 90 µg/mL gentamicin, from Sigma-Aldrich, Schnelldorf, Germany) for in vitro cultivation. To standardize the isolation of lymphocytes from lung tissue, we decided to sample a defined region of the lung, independently of the presence of lesions. The dorsal portion of the left caudal lobe was selected for this purpose, since the caudal lobes have been described to be a common site for APP lesions [33] but the right lung was washed for the collection of BALF.

### Histopathological analysis of lung tissue

Lung samples for histopathological analysis from acutely and chronically infected animals were fixed in neutral buffered formalin and embedded in paraffin wax. Tissue slides were routinely stained with hematoxylin and eosin and examined by a pathologist blinded to the different treatment groups. The samples were located adjacent to tissue used for lymphocyte isolation. Histopathological lesions associated with porcine pleuropneumonia, such as tissue necrosis, neutrophilic, histiocytic and lymphocytic infiltration, vascular leakage (including edema, bleeding, and fibrin in tissue or air spaces) and fibroplasia were graded (0 = not present, 1 = low grade, 2 = moderate grade, 3 = high grade).

### In vitro stimulation of lymphocytes

Freshly isolated cells from lung, blood, tracheobronchial lymph nodes and tonsils were stimulated in vitro with APP-CCE (4 µg/mL) for 18 h at 37 °C in 5% CO_2_. Cells were cultured in round-bottomed 96-well plates, at 5 × 10^5^ cells per well, in a volume of 200 µL. Four hours prior to harvesting the cells, Brefeldin A (BD GolgiPlug™, BD Biosciences, San Jose, CA, USA) was added at a final concentration of 1 µg/mL. In parallel, cells incubated in cell culture medium only served as a negative control. As a positive control for cytokine production, a further set of cells was cultivated in cell culture medium overnight but stimulated with phorbol 12-myristate 13-acetate (PMA; 50 ng/mL; Sigma-Aldrich) and Ionomycin (500 ng/mL; Sigma-Aldrich) during the last 4 h of incubation.

### Intracellular cytokine staining and FCM analysis

For FCM staining, cells were harvested and resuspended in PBS (without Ca^2+^/Mg^2+^) supplemented with 3% FCS. Monoclonal antibodies (mAbs) and secondary reagents that were used for cell surface staining and subsequent intracellular cytokine staining are listed in Table 1. Staining was performed in 96-well round-bottom plates with all incubation steps lasting for 20 min at 4 °C. For discrimination of dead cells, Live/Dead^®^ Near-IR stain kit (Invitrogen) was used. To fix and permeabilize the cells, BD Cytofix/Cytoperm and BD Perm/Wash (BD Biosciences, CA, USA) was employed according to manufacturer’s instructions.

**Table 1:** **Antibody panels**

Antigen	Clone	Isotype	Fluorochrome	Labelling strategy	Source of primary Ab
Intracellular cytokine staining for IL-17A and TNF-α					
CD4	74-12-4	IgG2b	PerCP-Cy5.5	Directly conjugated	BD Biosciences
CD8α	11/295/33	IgG2a	Pe-Cy7	Secondary antibody^a^	In house
TCR-γδ	PPT16	IgG2b	BV421	Biotin–streptavidin^b^	In house
IL-17A	SCPL1362	IgG1	Alexa647	Directly conjugated	BD Biosciences
TNF-α	MAb11	IgG1	BV605	Directly conjugated	BioLegend
Intracellular cytokine staining for IL-10					
CD4	74-12-4	IgG2b	Alexa647	Secondary antibody^c^	In house
CD8α	11/295/33	IgG2a	BV421	Biotin–streptavidin^b^	In house
IL-10	945A 4C4 37B1	IgG1	PE	Secondary antibody^d^	Invitrogen

^a^Goat Anti-Mouse IgG_2a_-PE-Cy7, SouthernBiotech.

^b^Brilliant Violet 421™ Streptavidin, BioLegend.

^c^Goat Anti-Mouse IgG_2b_-AlexaFluor647, Invitrogen.

^d^Goat Anti-Mouse IgG_1_-PE, SouthernBiotech.

FCM samples were analyzed on a FACSCanto™ II flow cytometer (BD Biosciences) equipped with three lasers (405, 488 and 633 nm). For automatic calculation of the compensation, single-stain samples were prepared. Between 5 × 10^5^ and 1 × 10^6^ lymphocytes were recorded per sample. Gating of the lymphocytes, doublet discrimination and dead-cell exclusion were performed for all samples as displayed in Additional file 2. Data were processed by FACSDiva software (Version 6.1.3 BD Biosciences) and transferred to Microsoft Excel (Office 2010; Microsoft, Redmond, WA, USA) for further calculations and preparation of graphs.

### Statistical analysis

Spearman’s rank correlation test was used to investigate the correlation between the frequency of Th17 cells and disease parameters in individual pigs. Spearman’s rank correlation coefficients (ρ) and corresponding *p* values were calculated in SPSS software (2011, IBM, SPSS Statistics for Windows, Version 20.0, Armonk, NY, IBM Corp.). SPSS was also applied to produce correlation graphs. For further elaboration of graphs, Inkscape (Version 0.91; Free and Open Source Software licensed under the GPL) was used.

## Results

### Establishment of an infection model for APP subclinical infection

**Figure 1:**
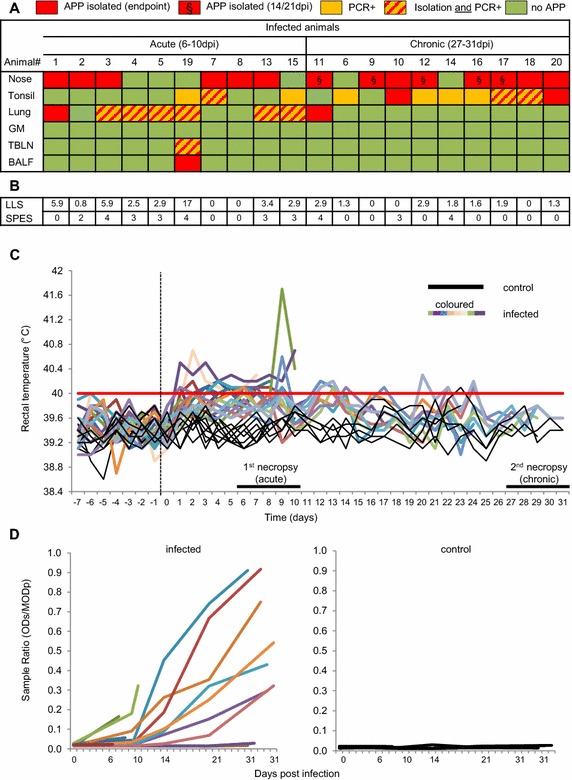
**Microbiological investigation, lung pathology, clinical signs and antibody titers of APP-infected pigs. A** The presence of APP was investigated at different host locations during necropsy (GM, *Glandula mandibularis*; TBLN, tracheobronchial lymph node; BALF, bronchoalveolar lavage fluid). Red boxes indicate APP detection by agar isolation, orange boxes indicate APP detection by PCR and orange boxes with red lines indicate positive results by both techniques. Green boxes indicate negative findings for APP. Results shown in the table refer to sampling on the day of euthanasia. The nasal swabs of animals #11, 9, 12, 16 and 17 were tested positive only on day 14 and/or 21 pi. This is indicated by red boxes with §. **B** Pathology of the lung was assessed by lung lesion score (LLS) for the lung tissue and by slaughterhouse pleurisy evaluation system (SPES) for the pleura. **C** Rectal temperatures were measured daily in both infected (colored lines) and control (black lines) animals. Body temperature of 40 °C or higher was considered as fever (red line). **D** Humoral response against APP serotype 2. Data are expressed as a ratio between optical density of the sample (ODs) and the mean of the optical density of the positive control (MODp). Colored lines in the left graph show ratios for infected animals, black lines in the right graph indicate ratios from sera of control animals.

To confirm the establishment of a subclinical APP infection in the pigs of our study, bacteriological and clinical parameters were investigated (Figure 1). APP could be isolated from the nose of the majority of infected animals both from the acute (6–10 dpi) and from the chronic period (27–31 dpi) (Figure 1A). Animals belonging to the latter group were additionally tested at 14 and 21 dpi, for weekly monitoring. The nasal swabs of some of these animals were positive during weekly monitoring but not at the time of death (indicated by a § in Figure 1A). Isolation of APP from tonsils was often impaired by overgrowth of contaminating flora, but for the majority of animals from the chronic phase (8 out of 10) APP could be identified by PCR. At the early endpoint (6–10 dpi), the location from which APP was most frequently isolated was the lung, with 7 positive samples out of 10. In contrast, at the late endpoint (27–31 dpi) only 1 out of 10 samples was positive in the lung. APP was detected in BALF and TBLN of only one animal, which died suddenly at 8 dpi (#19). APP could not be detected in salivary glands (*G. mandibularis*, GM) of any animal.

Macroscopically visible lung tissue alterations were highly variable, which is partially reflected by the lung lesion and SPES scores (Figure 1B; Additional file 3 for representative animals). On average, LLS scores from animals sacrificed during the acute phase were higher than during the chronic phase. Typical histopathological findings indicating porcine pleuropneumonia were found in infected pigs varying from acute (hyperemia, edema, neutrophilic infiltration) to chronic (sequestration of necrotic areas by a mixed inflammatory infiltrate and fibroplasia) inflammatory tissue alterations (Additional file 4).

To evaluate the health status of the pigs, rectal temperature was measured daily together with other clinical parameters. Generally, infected animals developed clinical symptoms like dyspnea and coughing and were more lethargic than controls within the first 10 dpi. No significant differences in average daily weight gain were observed between infected (mean 724.7 g ± standard deviation of 92.6 g) and control (785.3 g ± standard deviation of 130.7 g) animals during the first 3 weeks post infection. Body temperature of infected animals started rising towards 40 °C immediately after the day of infection (day 0), with some animals reaching high temperature levels within 1–10 dpi (Figure 1C). Then, the rectal temperature stabilized between 39.5 and 40 °C for 2 weeks to finally align with the levels of control animals at the end of the study period (25–31 dpi). In parallel, the APP 2-specific humoral response was evaluated throughout the experiment (Figure 1D). Most of the infected animals produced antibodies against APP 2 after 14 days, i.e., antibodies were only detectable in animals that survived until the chronic phase of infection. No APP 2 antibodies were detectable in control animals at any time point.

### Production of IL-17A and/or TNF-α by CD4^+^ T cells in response to APP-CCE

**Figure 2:**
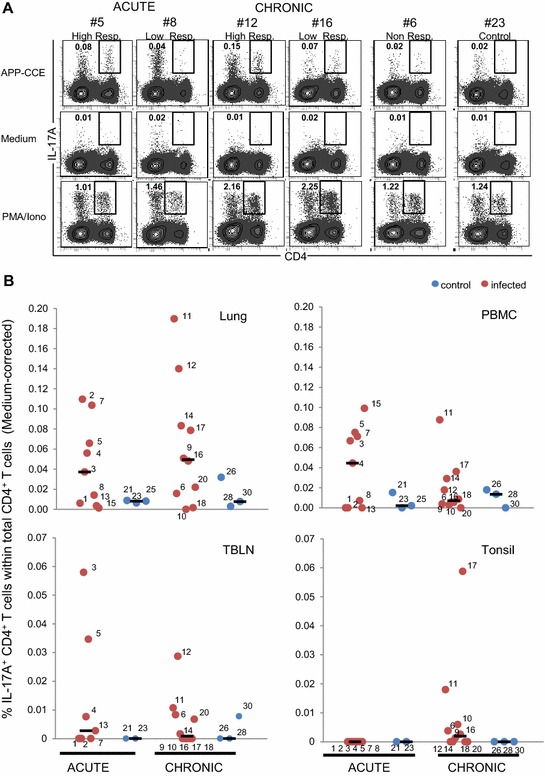
**APP-CCE specific IL-17A-producing CD4** ^**+**^ **T cells in lung, blood, tracheobronchial lymph nodes and tonsils.** Cells isolated from lung, blood (PBMC), tracheobronchial lymph nodes (TBLN) and tonsils were incubated overnight with APP crude capsular extract (APP-CCE), medium or PMA/Ionomycin. Living lymphocytes were gated (not shown; see Additional file 1) and further analyzed for the expression of IL-17A and CD4. **A** For the lung, data from representative animals from different groups are displayed: #5 and #8 for the acute phase, designated as “high responder” and “low responder” respectively; #12 and #16 for the chronic phase designated as “high responder” and “low responder” respectively; #6, designated as non-responder and control #23. Approximately 1 × 10^6^ (APP and medium) and 2 × 10^5^ (PMA/Ionomycin) cells are shown in the contour plots. Numbers displayed within the contour plots indicate the percentages of IL-17A^+^ CD4^+^ T cells within total CD4^+^ T cells. **B** Frequency of IL-17A^+^ CD4^+^ T cells within total CD4^+^ T cells in lung, blood (PBMC), tracheobronchial lymph node (TBLN) and tonsils of all infected animals (red dots) and control animals (blue dots) during acute and chronic phase. Numbers next to colored dots indicate numbers of individual animals. Median percent values are indicated by black bars. Medium-corrected percent values are presented (% of IL-17A^+^ CD4^+^ T cells within total CD4^+^ T cells for APP-CCE stimulation minus % of IL-17A^+^ CD4^+^ T cells within total CD4^+^ T cells for medium incubation).

For characterization of the T-cell mediated immune response, freshly isolated cells from lung, PBMCs, TBLN and tonsils were subjected to in vitro stimulation with APP-CCE of APP 2 followed by ICS for IL-17A and TNF-α. Medium- and PMA/Ionomycin-stimulated cultures served as negative and positive controls, respectively. Total living lymphocytes were gated and analyzed for CD4 expression and IL-17A production (Additional files 2A–D). A considerable variability in frequencies of IL-17A^+^ CD4^+^ T cells was found between different animals and organs following APP-CCE stimulation (Figure 2). Figure 2A shows representative data of IL-17A production and CD4 expression in lung-derived lymphocytes isolated from different animals during the acute and chronic phase. For each time point, original FCM data from one animal with a high and a low frequency of APP-CCE-responsive IL-17A^+^ CD4^+^ T cells is shown. In addition, respective contour plots for lymphocytes from an APP-infected but apparently non-responding animal (frequency of IL-17A^+^ CD4^+^ T cells was higher or at the same level for medium stimulation as compared to APP-CCE stimulation) as well as an animal from the control group are presented. Following PMA/Ionomycin stimulation IL-17A production was found in a subpopulation of CD4^+^ T cells from all animals. Similar findings were obtained in PBMC cultures, albeit frequencies of IL-17A^+^ CD4^+^ T cells were somewhat lower (Additional file 5). Overall, the highest number of animals with elevated frequencies of APP-CCE reactive-IL-17A^+^ CD4^+^ T cells was found within lung and PBMCs (Figure 2B). During the acute phase the frequencies of IL-17A^+^ CD4^+^ T cells in the lung were substantially higher in five out of nine animals compared to the control animals. This was similar during the chronic phase, with two animals (#11, #12) showing even increased frequencies of IL-17A^+^ CD4^+^ T cells. Within PBMCs the number of responding animals was similar to the lung during the acute phase but, later in the chronic phase, the median of the frequency of IL-17A^+^ CD4^+^ T cells dropped down to control levels. In TBLN and tonsils, CD4^+^ T cells of only a few infected animals responded with IL-17A production to APP-CCE stimulation, regardless of the time post infection (Figure 2B).

**Figure 3:**
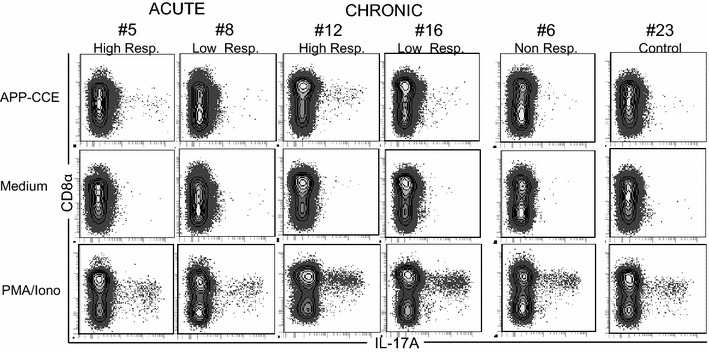
**Expression of CD8α by IL-17A** ^**+**^ **CD4** ^**+**^ **T cells in the lung.** Cells isolated from lung tissue were incubated overnight with APP crude capsular extract (APP-CCE), medium or PMA/Ionomycin and subsequently analyzed for CD4, CD8α and IL-17A expression by FCM. Living CD4^+^ T cells were gated (not shown; see Additional file 1) and subsequently investigated for expression of CD8α and IL-17A. Data from the same animals as in Figure 2 are shown. Approximately 1 × 10^5^ (APP and medium) and 5 × 10^4^ (PMA/Ionomycin) CD4^+^ T cells are shown in the contour plots.

Expression of CD8α is correlated with activation and/or memory formation of porcine CD4^+^ T cells [34] and following PMA/Ionomycin stimulation, IL-17A producing CD4^+^ T cells mainly have a CD8α^+^ phenotype [17]. We therefore analyzed CD8α expression and IL-17A production in gated CD4^+^ T cells following APP-CCE stimulation (Figure 3; Additional file 6). In the lungs of animals belonging to the acute group, the majority of IL-17A^+^ CD4^+^ T cells following APP-CCE and PMA/Ionomycin stimulation were CD8α^dim^, whereas in the chronic phase CD8α expression tended to be higher and this applied equally to APP-CCE and PMA/Ionomycin stimulated cells (Figure 3). Similar results were obtained with PBMCs albeit here an up-regulation of CD8α in IL-17A-producing CD4^+^ T cells from the acute to the chronic phase was less obvious (Additional file 6).

**Figure 4:**
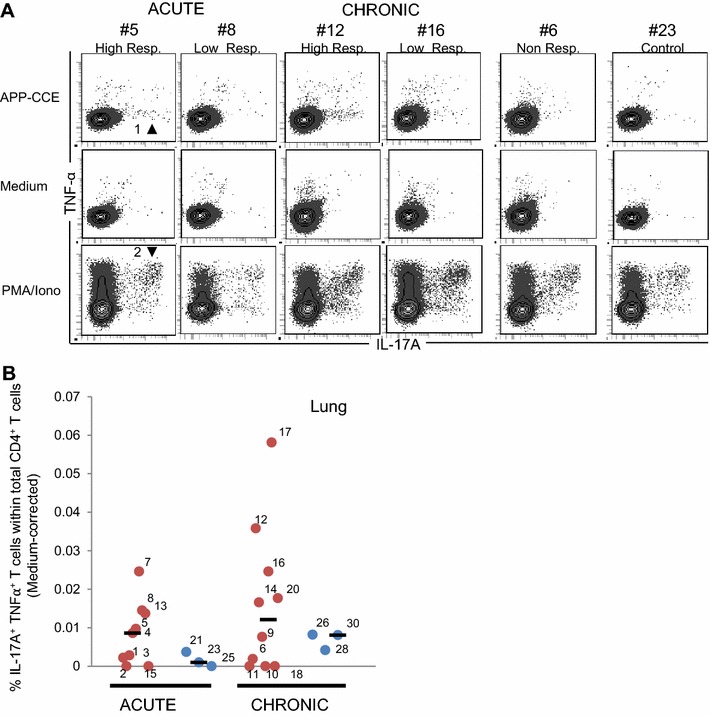
**Co-production of TNF-α and IL-17A by CD4** ^**+**^ **T cells in the lung.** Phenotyping and intracellular cytokine staining were performed on cells from lung tissue following overnight in vitro stimulation (APP-CCE, medium, PMA/Ionomycin). **A** Living CD4^+^ T cells were gated (not shown; see Additional file 1) and further analyzed for production of TNF-α and IL-17A. Data from the same animals as in Figure 2 are shown. Approximately 1 × 10^5^ (APP and medium) and 5 × 10^4^ (PMA/Ionomycin) cells are shown in the contour plots. **B** Frequency of IL-17A/TNFα co-producing CD4^+^ T cells in lung tissue of infected animals (red dots) and control animals (blue dots) during acute and chronic phase. Numbers next to colored dots indicate numbers of individual animals. Median percent values are indicated by black bars. Medium-corrected percent values are presented (% of IL-17A^+^ TNF-α^+^ cells within total CD4^+^ T cells for APP-CCE stimulation minus  % of IL-17A^+^ TNF-α^+^ cells within total CD4^+^ T cells for medium-incubation). Arrow heads are introduced in the main text.

Next, we analyzed co-production of IL-17A and TNF-α within APP-CCE-stimulated CD4^+^ T cells (Figure 4; Additional file 7; for gating strategy see Additional files 2E and F), since frequent co-production of TNF-α has been observed in PMA/Ionomycin-stimulated porcine IL-17A^+^ CD4^+^ T cells [17]. The majority of IL-17A-producing CD4^+^ T cells isolated from lung tissue (Figure 4) or within PBMC (Additional file 7) did not co-produce TNF-α following APP-CCE stimulation (Figure 4A; Additional file 7A, indicated by arrowhead 1). In contrast, but in accordance with previously published data [17] following PMA/Ionomycin stimulation most IL-17A^+^ CD4^+^ T cells co-produced TNF-α (Figure 4A; Additional file 7A, arrowhead 2). Despite low frequencies for the majority of APP-infected animals, a tendency of higher frequencies of TNF-α^+^ IL-17A^+^ CD4^+^ T cells was found within lymphocytes from APP-infected pigs compared to control animals following APP-CCE stimulation. This applied to cells isolated from lung tissue (Figure 4B) and blood (Additional file 7B).

### APP-CCE stimulation induced production of IL-17A by a subset of lymphocytes that is neither TCR-γδ^+^ nor CD4^+^

**Figure 5:**
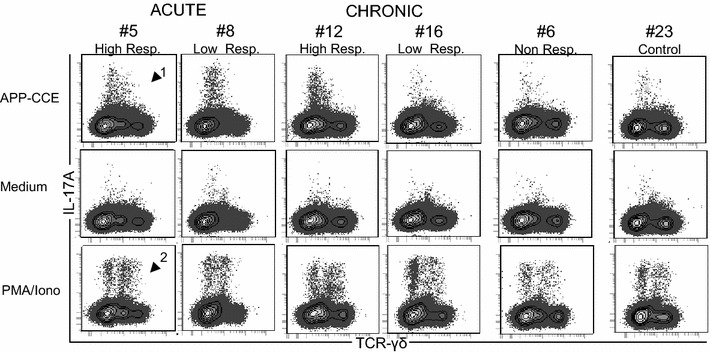
**Production of IL-17A by non-CD4** ^**+**^ **cells and γδ T cells in the lung.** Cells isolated from lung tissue were incubated overnight with APP-CCE, medium or PMA/Ionomycin and subsequently analyzed for CD4, TCR-γδ and IL-17A expression by FCM. Living lymphocytes excluding CD4^+^ T cells (not shown; see Additional file 1) were gated and further analyzed for expression of IL-17A and TCR-γδ. Data from the same animals as in Figure 2 are shown. Approximately 1 × 10^6^ (APP and medium) and 2 × 10^5^ (PMA/Ionomycin) cells are shown in the contour plots. Arrow heads are introduced in the main text.

After stimulation with APP-CCE, at least for some animals a considerable number of CD4^−^ cells showed the ability to produce IL-17A in parallel to CD4^+^ T cells (Figure 2A). This finding was more prominent in the lung, but it was detected also in PBMCs (Additional file 5). Since porcine γδ T cells were previously identified as potential IL-17A producers following PMA/Ionomycin stimulation [16], we hypothesized that these cells might also contribute to IL-17A production following APP-CCE stimulation. Hence, CD4^−^ cells were further gated for expression of TCR-γδ and IL-17A (Additional files 2E and G). Interestingly, these IL-17A-producing cells induced by APP-CCE stimulation were TCR-γδ^−^ (Figure 5; Additional file 8, arrowhead 1). However, they were also present in lymphocytes isolated from control animals (Figure 5; Additional file 8, last column), which seems to indicate that these cells did not require a preceding in vivo priming by APP. Their frequency was quite variable between individual animals but mostly exceeded the very low frequency of IL-17A-producing CD4^−^ TCR-γδ^−^ cells identified in medium stimulated cultures. Following PMA/Ionomycin stimulation, IL-17A-producing γδ T cells could be identified (Figure 5; Additional file 8, bottom panel, arrowhead 2), although IL-17A producing cells isolated from lung tissue showed a dim expression of TCR-γδ. Overall, this confirms the potential of porcine γδ T cells for IL-17A production but our data suggests that APP-CCE stimulation does not induce IL-17A production in this prominent porcine T-cell subset.

### Inconsistent IL-10 production by CD4^+^ T cells following APP-CCE stimulation

**Figure 6:**
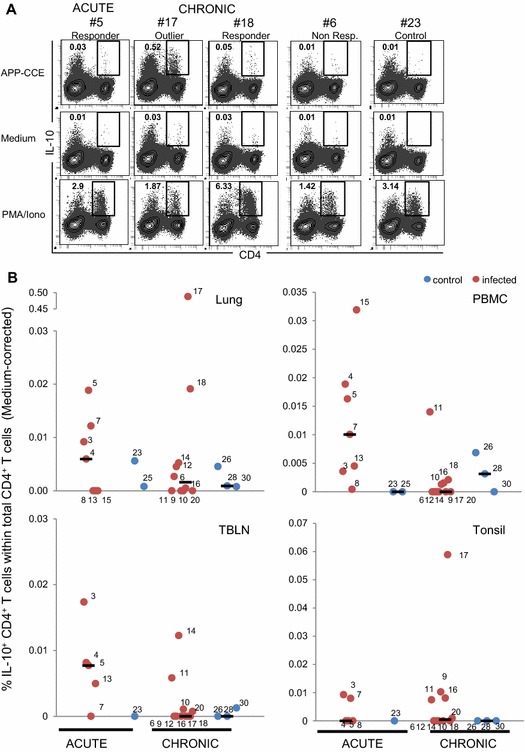
**APP-CCE specific IL-10-producing CD4** ^**+**^ **T cells in lung, peripheral blood, tracheobronchial lymph nodes and tonsils.** Cells isolated from lung, blood (PBMC), tracheobronchial lymph nodes (TBLN) and tonsils were incubated overnight with APP-CCE, medium or PMA/Ionomycin and subsequently analyzed by FCM. Living cells were gated (not shown; see Additional file 1) and further analyzed for expression of IL-10 and CD4. **A** For the lung, data from representative animals from different groups are displayed: #5 for the acute phase, designated as “responder”; #17 and #18 for the chronic phase, designated as “outlier” and “responder”, respectively; #6, designated as “non-responder” and control pig #23. Approximately 5 × 10^5^ (APP and medium) and 1.5 × 10^5^ (PMA/Ionomycin) cells are shown in the contour plots. Numbers displayed within the contour plots indicate the percentage of IL-10^+^ CD4^+^ T cells within total CD4^+^ T cells. **B** Frequency of IL-10^+^ CD4^+^ T cells in lung, PBMC, TBLN and tonsils of infected animals (red dots) and control animals (blue dots) during acute and chronic phase. Numbers next to colored dots indicate numbers of individual animals. Median percent values are indicated by black bars. Medium-corrected percent values are presented (% of IL-10^+^ CD4^+^ T cells within total CD4^+^ T cells for APP-CCE stimulation minus % of IL-10^+^ CD4^+^ T cells within total CD4^+^ T cells for medium incubation).

To investigate a potential induction of IL-10-producing lymphocytes in our APP-CCE in vitro stimulation assay the production of IL-10 was analyzed in parallel samples to that of IL-17A/TNF-α in combination with cell surface staining for CD4 and CD8α expression. Overall, with the exception of cells isolated from the APP-infected pig #17, frequencies of IL-10^+^ CD4^+^ T cells were low and inconsistently distributed between individual animals during the acute and the chronic phase as well as different organs (Figure 6). However, in cells of some animals, the frequency of IL-10^+^ CD4^+^ T cells was at least twofold higher following APP-CCE stimulation compared to medium and was also higher compared to that from control animals. This applied to animals #3, 5 and 7 in the lung during the acute phase and animal #18 during the chronic phase (see also Figure 6A for original FCM data). Similarly, within PBMCs, animals #7, 4, 5, 11 and 15 appeared to have APP-CCE-reactive IL-10^+^ CD4^+^ T cells above background levels (see also Additional file 9 for original FCM data). Isolated during the chronic phase, CD4^+^ T cells from animal #17 showed an exceptionally high frequency of IL-10 producing cells after APP-CCE stimulation, both in lung and in tonsils (Figures 6A and B). The reasons for this are unknown. CD4^−^ IL-10-producing cells were identified (Figure 6A; Additional file 9 and data not shown) but similar frequencies were found for APP-CCE- and medium-stimulated cultures.

### Frequency of IL-17A^+^ CD4^+^ T cells correlates positively with disease parameters during the chronic phase of infection

**Figure 7:**
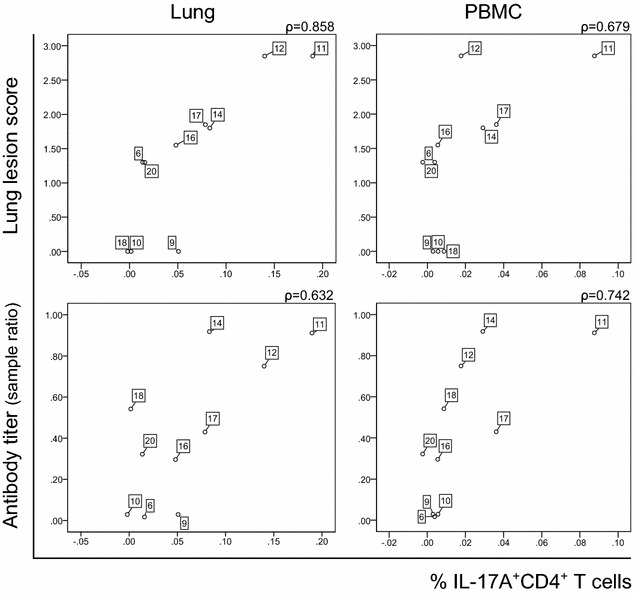
**Correlation of the frequency of IL-17A** ^**+**^ **CD4** ^**+**^ **T cells with lung lesion score and antibody titers during the chronic phase.** Scatterplots show correlation of the frequency of IL-17A^+^ CD4^+^ T cells isolated from lung and blood with lung lesion score (top panel) and antibody titer (bottom panel) in chronically infected animals. Antibody titer is expressed as a ratio between optical density of the sample (ODs) and the mean of the optical density of the positive control (MODp). Spearman’s Rank Correlation coefficients (ρ) are displayed above each scatterplot.

We next investigated whether the variable frequency of APP-CCE-reactive IL-17A^+^ CD4^+^ T cells between different animals and organs correlated with parameters of APP pathogenesis and also APP-specific antibody titers. In chronically infected animals, the frequency of APP-CCE-reactive IL-17A^+^ CD4^+^ T cells isolated from the lung showed a positive correlation with LLS (Spearman’s rho = 0.858; *p* = 0.001) and with APP 2 antibody titers (Spearman’s rho = 0.632, *p* = 0.05). For IL-17A^+^CD4^+^ T cells within PBMCs of the same group of animals also a positive correlation with LLS and antibody titers was found (Spearman’s rho of 0.679; *p* = 0.031 and 0.742, *p* = 0.014 respectively) (Figure 7). In contrast, for acutely infected animals no positive correlation of IL-17A^+^ CD4^+^ T cells isolated from lung or blood with the LLS was found (Additional file 10). Additionally, the frequencies of APP-CCE-reactive IL-17A^+^ CD4^+^ T cells isolated from the lung and blood were tested for correlation with histological scores of the lung tissue adjacent to tissue used for isolation of lymphocytes (Additional file 11). A total histological score for each sample was calculated by summing up all analyzed parameters (see Additional file 4) and was used to calculate the correlation. No significant correlation was found, which may indicate that APP-CCE-reactive IL-17A^+^ CD4^+^ T cells have a general capacity for lung homing.

## Discussion

The main focus of the study was to characterize the cytokine response of T cells isolated from pigs undergoing either an acute or a subclinical APP infection. To address this question, we first aimed to establish an infection model that evokes typical but not lethal APP disease symptoms and induces a status of subclinical infection. Most of the experimental infections described in the literature so far focused on studying the acute phase of APP infection [35–37]. The outcome of APP infection is depending on the route of infection, the dose and the virulence of the strain [38]. Baarsch et al. [39] demonstrated that the route of infection influences the distribution of lung lesions, with the intranasal inoculation provoking mainly unilateral lesions and the endotracheal infection inducing diffuse bilateral pneumonia. To better mimic natural infection, we decided to perform an intranasal inoculation. To avoid loss of bacterial solution by swallowing or miscalculation of the actual infection dose by aerosol administration, the inoculum was sprayed directly into the nostrils rather than deposited as a liquid. Considering that vaporizing the bacterial solution into finely atomized particles increases the chance of APP reaching the alveoli [1], the infection dose was kept to low levels to avoid the induction of a per-acute form. The endpoints where chosen to compare acute and subclinical phase of infection. We hypothesized that at days 6–10 a first activation of T cells may be measurable like previously reported for acute influenza A virus infection [40]. Additionally, the median duration of APP tonsillar colonization was reported around 7–8 weeks post-infection (pi) [41] leading to the assumption that APP would still be present at days 27–31 pi causing a subclinical infection. Pathological and clinical findings confirmed the establishment of a bi-phasic course of infection. The variety of rectal temperatures obtained during the acute phase paralleled with the variety of lung lesions observed at necropsy, with patterns ranging from severe and diffuse pneumonia with dark red–purple areas (per-acute), localized necrotizing pneumonia accompanied by fibrinous pleurisy (acute) to firm adhesive pleurisy and organized sequestra (chronic). On the opposite, during the chronic phase animals showed more uniform lesions and their body temperatures dropped down to physiological levels. These results indicate that our infection model successfully induced an acute infection that resolved into a subclinical one. Furthermore, this dichotomy could also be found by analyzing the presence of APP at different host compartments. During the acute phase, APP was mostly detected in the lung (7 out of 10), and hardly in the tonsils (3 out of 10). During the chronic phase, only one animal was positive in the lung, whereas in the tonsils 8 out of 10 tested positive. This might indicate a shift in the tropism of the bacteria from the lower to the upper respiratory tract and might be interpreted as an attempt of the bacteria to escape from the local immune response in the lung.

In frame of this study, we developed an in vitro stimulation assay using a crude capsular extract of APP. This assay was used to investigate the production of IL-17A, TNF-α and IL-10 in lymphocytes isolated from lung, peripheral blood, tracheobronchial lymph node and tonsil. Our data suggest that IL-17A-producing CD4^+^ T cells are induced in the lung tissue and blood of most APP-infected pigs. IL-17 is a pro-inflammatory cytokine, known to play a role in pulmonary infection and neutrophil recruitment [42, 43]. Its role in veterinary animal species [44] and its up-regulation in the surroundings of APP colonies in affected lung lesions on mRNA level have been previously described [14]. Thus it is tempting to speculate that the APP-CCE-reactive IL-17A producing CD4^+^ T cells identified in this study represent APP-specific Th17 cells. The specificity of these cells is corroborated by two findings. First, similar to PMA/Ionomycin-induced IL-17A^+^ CD4^+^ T cells, these putative APP-specific Th17 cells expressed low or intermediate levels of CD8α. Its expression in porcine CD4^+^ T cells is related to activation and/or memory formation [34, 45]. Therefore, this CD8α expression can be interpreted as an indication that APP-CCE-reactive IL-17A-producing CD4^+^ T cells performed an in vitro recall response. Secondly, APP-CCE reactive IL-17A producing CD4^+^ T cells were nearly completely absent in lymphocytes isolated from control pigs, indicating that APP-naive CD4^+^ T cells did not respond to in vitro stimulation with APP-CCE.

Human [46] and porcine [17] CD4^+^ T cells have the capacity to co-produce IL-17A and TNF-α following PMA/Ionomycin stimulation. Furthermore, a synergistic effect between IL-17 and TNF-α has been reported, enhancing neutrophil migration [47]. Also, TNF-α is known to play a major role in the immune-pathogenesis of APP infection [35, 48]. Following APP-CCE stimulation, we found that the majority of CD4^+^ T cells that produced IL-17A did not co-produce TNF-α. This suggests that CD4^+^ T cells are not a main source of TNF-α during APP infection.

Interestingly, a small subset of CD4^−^ TCR-γδ^−^ cells showed production of IL-17A upon APP-CCE stimulation but was also identified in control animals. Innate sources of IL-17 are described [49] such us iNKT cells [50], NK cells [51] and innate lymphoid cells type 3 (ILC3) [52]. To affirm that the population revealed in our study is actually belonging to one of the subsets mentioned above, further investigations would be needed.

For several persistent pathogens like *Mycobacterium tuberculosis* [21], *Leishmania* spp. [23] and *Toxoplasma gondii* [22], IL-10 has been reported to impair their clearance by influencing the delicate balance between suppression and activation of host immune responses. In this study we therefore evaluated the production of IL-10 by CD4^+^ T cells in different organs but a specific induction upon APP-CCE stimulation was found only in few animals and frequencies of IL-10^+^ CD4^+^ T cells were rather low. In a previous study, IL-10 mRNA was predominantly found within lung lesions of APP-infected pigs but was only minimal in non-affected areas. [53]. Of note, lung tissue samples in our study were derived from a defined anatomic area (dorsal portion of the left caudal lobe) and only in a single animal (#11) sequestra were included in that area. The frequency of IL-10-producing CD4^+^ T cells derived from this animal was high in both APP-CCE-(0.17%) and medium-(0.17%) stimulated cultures, resulting in a medium-corrected value of zero (Figure 6B, lung). Thus, we cannot exclude a potential role of IL-10 in the immune pathogenesis of APP infection. Further studies on affected lung lesions should be carried out in future studies to decipher the exact role of IL-10 in APP infections.

Finally, we observed that the frequency of Th17 cells in lung and PBMCs from chronically infected animals correlated positively with the lung lesion score and APP-specific antibody titers. Such a correlation was not found in the animals during the acute infection phase. This could be related to the enormous variety of lung lesions (diffuse/local necrotic and hemorrhagic areas, fibrosis, formation of sequestra, absence of lesions) observed at the necropsy during the acute phase, as described above. Moreover, no positive correlation between Th17 cells and the histological score of lung tissue samples from which lymphocytes had been isolated was found in the chronic phase of infection. Together with the positive correlation between frequency of Th17 cells in lung tissue as well as blood and the lung lesion score, this may indicate that these APP-specific Th17 cells have a general capacity for lung homing and also recirculate via the bloodstream. This would correspond with functional attributes ascribed to effector memory T cells [54]. However, the precise functional role of these Th17 cells in APP pathology and persistence remains speculative. It is well established that cytokine production by Th17 cells can have protective but also pathologic roles in lung immunity [19]. An excessive recruitment of neutrophils due to IL-17 production by CD4^+^ T cells could lead to progressive inflammation, which might explain the positive correlation between lung lesion and IL-17 production. Additionally, APP chronic lung lesions are usually characterized by fibroplasia [1] and IL-17 was shown to be involved in the occurrence and the development of pulmonary fibrosis in rats [55]. Nevertheless, our in vitro stimulation system does not allow a distinction between actively IL-17A-producing CD4^+^ T cells in vivo (at the time of isolation) and the re-stimulation of quiescent APP-specific Th17 memory cells upon a second exposure to the antigen. Therefore, the precise role of the putative APP-specific Th17 cells in APP immunity, identified in our study, remains to be elucidated.

In conclusion, our results support previous findings that T cells are involved in the immune response to APP infection. We could show for the first time that APP-specific T cells with functional attributes of Th17 cells are induced in most APP-infected animals, which during the chronic phase of infection seem to positively correlate with lung lesion formation. Thus, our findings highlight the relevance of detailed immunological studies addressing T-cell differentiation for a better understanding of host-pathogen interactions in APP. Moreover, our infection model provides a solid basis for such studies in a controlled setting. This will contribute to a better understanding of APP pathogenesis and persistence.

## Additional files

**Additional file 1.** **Clinical score protocol.** Clinical examinations were performed daily throughout the experiment. Alterations in behavior, gait, presence of respiratory symptoms (cough and dyspnea), and body temperature were assessed and scored on a scale from 0 to 4 based on the listed symptoms or traits.

**Additional file 2.** **FCM gating hierarchy.** Representative example of the FCM gating strategy used in this study. Data is derived from lung of animal #12 (APP-infected). (**A**) Lymphocytes were gated according to their light scatter properties. (**B**) A FSC-W/H gate coupled with a SSC-W/H gate was applied in order to exclude potential doublet cells. (**C**) Near-IR stain was used for Live/Dead discrimination. Only Near-IR negative cells (live cells) were included in the following analyses. (**D**) Co-expression of CD4 and IL-17A for identification of IL-17A^+^ CD4^+^ T cells. (**E**) Cells were further distinguished in either CD4^+^ or CD4^−^ T cells. (**F**) Within the CD4^+^ subpopulation, the production of IL-17A and TNF-α was investigated. (**G**) Within the CD4^−^ subpopulation, the expression of IL-17A and TCR-γδ was investigated.

**Additional file 3.** **Pathological findings in the lung of acutely and chronically infected animals.** Lungs from representative animals, one for the acute and one for the chronic phase, are shown. (**A**) Bilateral diffuse hemorrhagic pneumonia and fibrinous pleurisy in an acutely infected animal (#3). (**B**) Necrotic foci surrounded by scar tissue (sequestra) and adhesive pleurisy with evidence of firm adhesions between visceral and parietal pleura in a chronically infected animal (#11).

**Additional file 4.** **Histological evaluation of lung tissue from infected animals.** Lung tissue of the dorsal portion of left caudal lung lobe (adjacent to samples used for lymphocyte isolation) was taken from acutely and chronically infected animals. This tissue was paraffin embedded, stained with hematoxylin and eosin, and examined for presence and quantity of parameters A–H (see legend). The quantity and presence of each parameter were assessed by using a score from 0 to 3 (0 = not present, 1 = low grade, 2 = moderate grade, 3 = high grade). No sample in this study presented lesions of grade 3; therefore this grade is not shown in the legend.

**Additional file 5.** **APP-specific induction of IL-17A** ^**+**^ **CD4** ^**+**^ **T cells in peripheral blood.** PBMCs were incubated overnight with APP crude capsular extract (APP-CCE), medium or PMA/Ionomycin. Living lymphocytes were gated (not shown; see Additional file 1) and further analyzed for the expression of IL-17A and CD4. Data from representative animals from different groups are displayed: #5 and #4 for the acute phase, designated as “high responder” and “low responder” respectively; #11 and #17 for the chronic phase designated as “high responder” and “low responder” respectively; #6, designated as non-responder and control pig #23. Approximately 7 × 10^5^ (APP and medium) and 2 × 10^5^ (PMA/Ionomycin) cells are shown in the contour plots respectively. Numbers displayed within the contour plots indicate the percentage of IL-17A^+^CD4^+^ T cells within total CD4^+^ T cells.

**Additional file 6.** **Expression of CD8α by IL-17A** ^**+**^ **CD4** ^**+**^ **T cells in peripheral blood.** PBMCs were incubated overnight with APP crude capsular extract (APP-CCE), medium or PMA/Ionomycin. Living lymphocytes were gated (not shown; see Additional file 1) and further analyzed for the expression of CD8α and IL-17A. Data from the same animals as in Additional file 2 is shown. Approximately 3 × 10^5^ (APP and medium) and 5 × 10^4^ (PMA/Ionomycin) cells are shown in the contour plots.

**Additional file 7.** **Co-production of TNF-α and IL-17A by CD4** ^**+**^ **T cells in peripheral blood.** Phenotyping and intracellular cytokine staining were performed on PBMC following overnight in vitro stimulation (APP-CCE, medium, PMA/Ionomycin). (**A**) Living CD4^+^ T cells were gated (not shown; see Additional file 1) and further analyzed for production of TNF-α and IL-17A. Data from the same animals as in Additional file 2 are shown. Approximately 3 × 10^5^ (APP and medium) and 5 × 10^4^ (PMA/Ionomycin) cells are shown in the contour plots. (**B**) Frequency of IL-17A/TNF-α co-producing CD4^+^ T cells in PBMC of infected animals (red dots) and control animals (blue dots) during acute and chronic phase. Numbers next to colored dots indicate numbers of individual animals. Median percent values are indicated by black bars. Medium-corrected percentage values are presented (% of IL-17A^+^ TNF-α^+^ cells within total CD4^+^ T cells for APP-CCE stimulation minus % of IL-17A^+^ TNF-α^+^ cells within total CD4^+^ T cells for medium incubation). Arrow heads are introduced in the main text.

**Additional file 8.** **Production of IL-17A by non-CD4** ^**+**^ **cells and γδ T cells in the peripheral blood.** PBMC were incubated overnight with APP-CCE, medium or PMA/Ionomycin and subsequently analyzed for CD4, TCR-γδ and IL-17A expression by FCM. Living lymphocytes excluding CD4^+^ T cells (not shown; see Additional file 1) were gated and further analyzed for expression of IL-17A and TCR-γδ. Data from the same animals as in Additional file 2 are shown. Approximately 7 × 10^5^ (APP and medium) and 2 × 10^5^ (PMA/Ionomycin) cells are shown in the contour plots. Arrow heads are introduced in the main text.

**Additional file 9.** **APP-CCE-specific IL-10-producing CD4** ^**+**^ **T cells in peripheral blood.** PBMC were incubated overnight with APP-CCE, medium or PMA/Ionomycin and subsequently analyzed by FCM. Living cells were gated (not shown; see Additional file 1) and further analyzed for expression of IL-10 and CD4. Data from representative animals from different groups are displayed: #15 for the acute phase and #11 for the chronic phase, both designated as “responders”; #6, designated as “non-responder” and control pig #23. Approximately 8 × 10^5^ (APP and medium) and 1.5 × 10^5^ (PMA/Ionomycin) cells are shown in the contour plots. Numbers displayed within the contour plots indicate the percentage of IL-10^+^ CD4^+^ T cells within total CD4^+^ T cells.

**Additional file 10.** **Correlation of the frequency of IL-17A** ^**+**^ **CD4** ^**+**^ **T cells with lung lesion score during the acute phase.** Scatterplots show correlation of the frequency of IL-17A^+^ CD4^+^ T cells isolated from lung and blood with lung lesion score in acutely infected animals. Spearman’s Rank Correlation Coefficients (ρ) are displayed above each scatterplot.

**Additional file 11.** **Correlation of the frequency of IL-17A** ^**+**^ **CD4** ^**+**^ **T cells with histological score of lung tissue during the chronic phase.** Scatterplots show correlation of the frequency of IL-17A^+^ CD4^+^ T cells isolated from lung and blood during the chronic phase with the histological score of the lung tissue sampled adjacent to tissue used for lymphocyte isolation. Histological scores for each sample were calculated by summing up the grading of all parameters shown in Additional file 4. Spearman’s Rank Correlation Coefficients (ρ) are displayed above each scatterplot.
