# Supplementary material for: Frequency of Th17 cells correlates with the presence of lung lesions in pigs chronically infected with Actinobacillus pleuropneumoniae
Source: Vet Res. 2017 Feb 6;48:4. doi: 10.1186/s13567-017-0411-z (PMC5294905; doi:10.1186/s13567-017-0411-z)
Supplement: Supplementary file 1 — Additional file 1. Clinical score protocol. Clinical examinations were performed daily throughout the experiment. Alterations in behavior, gait, presence of respiratory symptoms (cough and dyspnea), and body temperature were assessed and scored on a scale from 0 to 4 based on the listed symptoms or traits. [file 13567_2017_411_MOESM1_ESM.pdf]

Behavior	Gait	Cough	Dyspnea	Temperature	Score
Physiologic	Physiologic	Absent	Absent	38.5-39.5	0
Lethargic	Laying down			39.6-40	1
Apathetic	Sitting-dog position	Present	More costal or abdominal	40.1-41	2
Stupors	Swinging			>41	3
Comatose	Unable to stand		Only costal or abdominal	<38.5	4
