# Supplementary figures and images for: Frequency of Th17 cells correlates with the presence of lung lesions in pigs chronically infected with Actinobacillus pleuropneumoniae
Source: Vet Res. 2017 Feb 6;48:4. doi: 10.1186/s13567-017-0411-z (PMC5294905; doi:10.1186/s13567-017-0411-z)

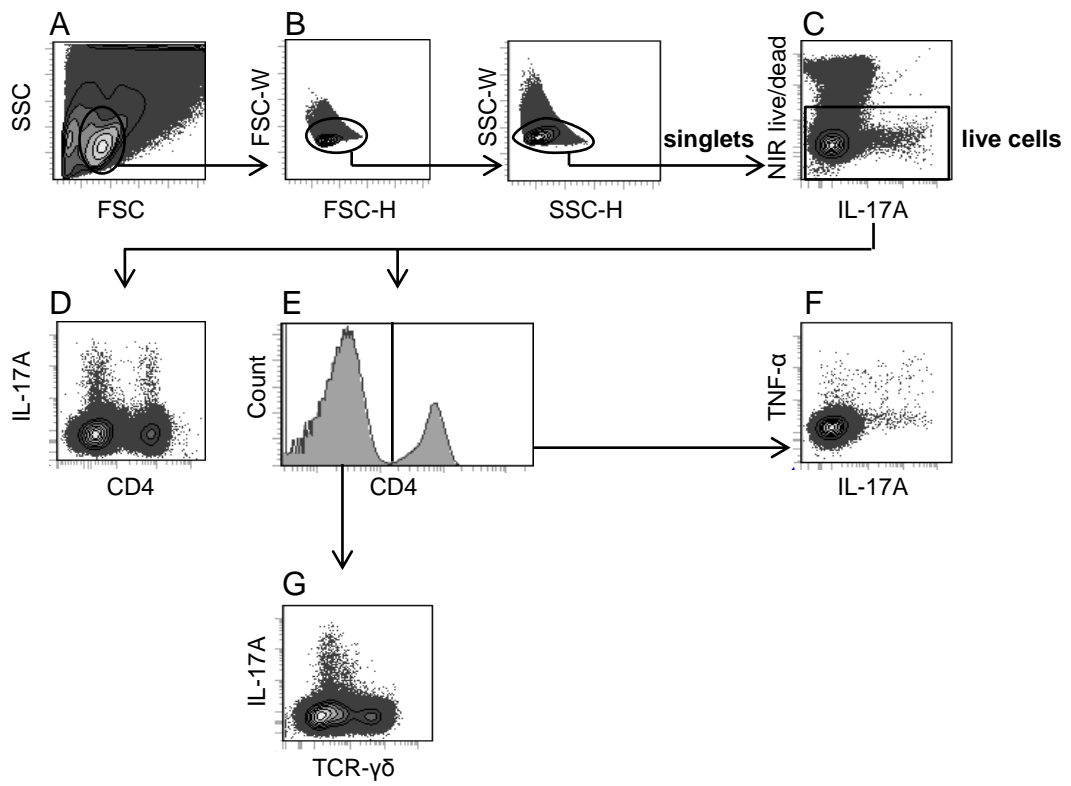

Supplement: Supplementary file 2 — Additional file 2. FCM gating hierarchy. Representative example of the FCM gating strategy used in this study. Data is derived from lung of animal #12 (APP-infected). (A) Lymphocytes were gated according to their light scatter properties. (B) A FSC-W/H gate coupled with a SSC-W/H gate was applied in order to exclude potential doublet cells. (C) Near-IR stain was used for Live/Dead discrimination. Only Near-IR negative cells (live cells) were included in the following analyses. (D) Co-expression of CD4 and IL-17A for identification of IL-17A+ CD4+ T cells. (E) Cells were further distinguished in either CD4+ or CD4− T cells. (F) Within the CD4+ subpopulation, the production of IL-17A and TNF-α was investigated. (G) Within the CD4− subpopulation, the expression of IL-17A and TCR-γδ was investigated. [file 13567_2017_411_MOESM2_ESM.pdf]

Acute

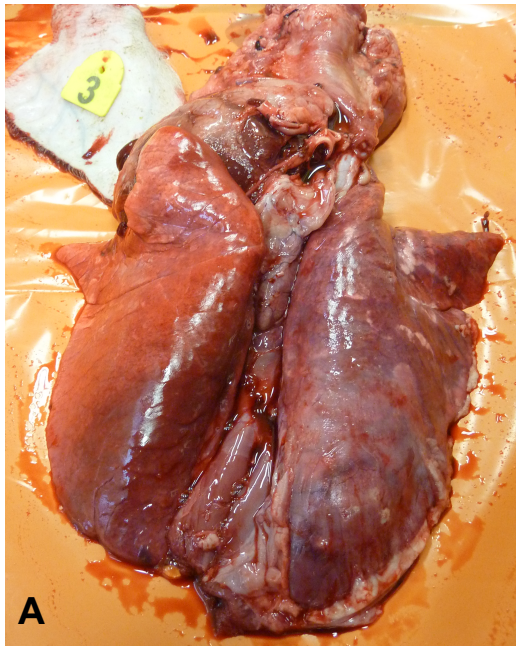

Chronic

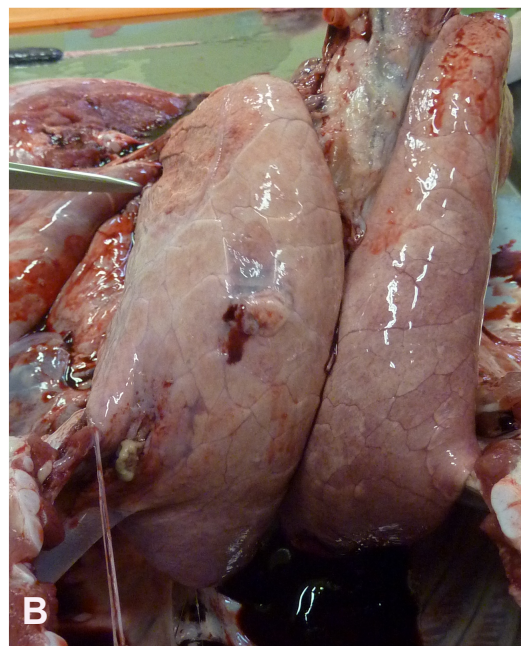

Supplement: Supplementary file 3 — Additional file 3. Pathological findings in the lung of acutely and chronically infected animals. Lungs from representative animals, one for the acute and one for the chronic phase, are shown. (A) Bilateral diffuse hemorrhagic pneumonia and fibrinous pleurisy in an acutely infected animal (#3). (B) Necrotic foci surrounded by scar tissue (sequestra) and adhesive pleurisy with evidence of firm adhesions between visceral and parietal pleura in a chronically infected animal (#11). [file 13567_2017_411_MOESM3_ESM.pdf]

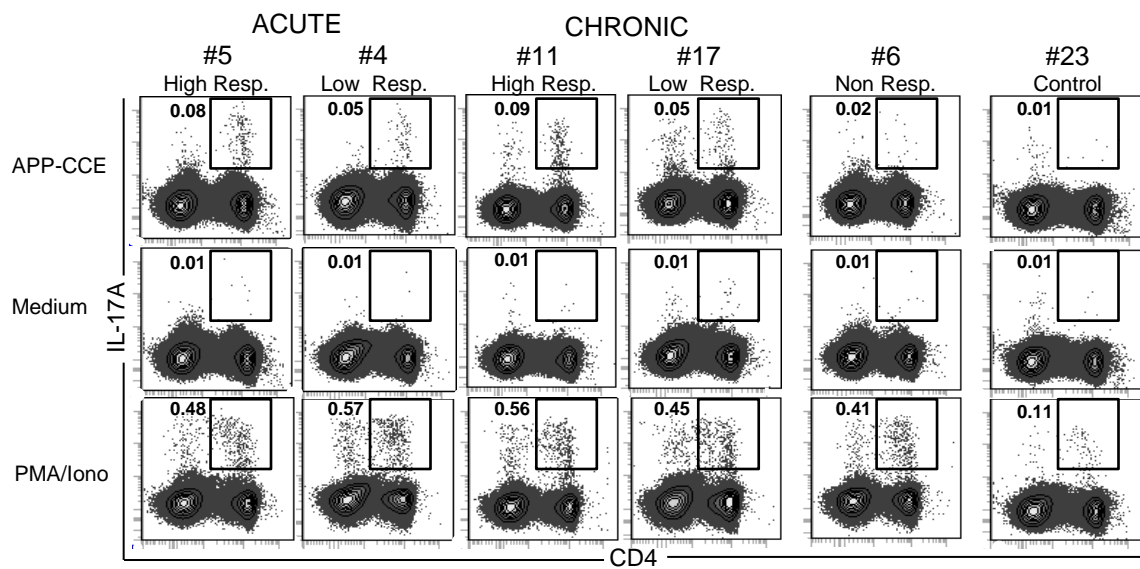

Supplement: Supplementary file 5 — Additional file 5. APP-specific induction of IL-17A + CD4 + T cells in peripheral blood. PBMCs were incubated overnight with APP crude capsular extract (APP-CCE), medium or PMA/Ionomycin. Living lymphocytes were gated (not shown; see Additional file 1) and further analyzed for the expression of IL-17A and CD4. Data from representative animals from different groups are displayed: #5 and #4 for the acute phase, designated as “high responder” and “low responder” respectively; #11 and #17 for the chronic phase designated as “high responder” and “low responder” respectively; #6, designated as non-responder and control pig #23. Approximately 7 × 10^5^ (APP and medium) and 2 × 10^5^ (PMA/Ionomycin) cells are shown in the contour plots respectively. Numbers displayed within the contour plots indicate the percentage of IL-17A+CD4+ T cells within total CD4+ T cells. [file 13567_2017_411_MOESM5_ESM.pdf]

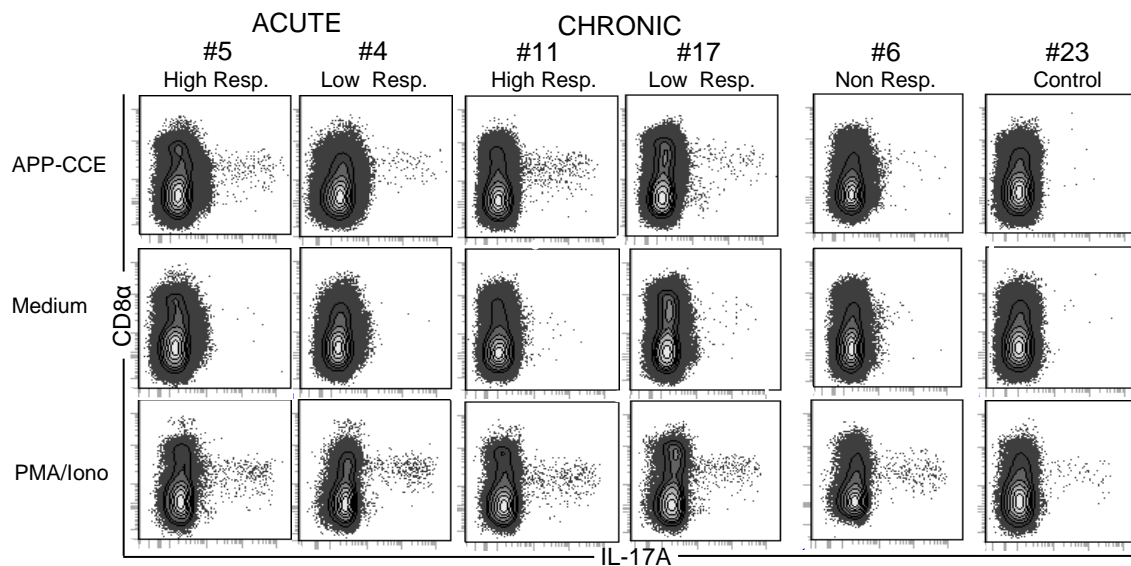

Supplement: Supplementary file 6 — Additional file 6. Expression of CD8α by IL-17A + CD4 + T cells in peripheral blood. PBMCs were incubated overnight with APP crude capsular extract (APP-CCE), medium or PMA/Ionomycin. Living lymphocytes were gated (not shown; see Additional file 1) and further analyzed for the expression of CD8α and IL-17A. Data from the same animals as in Additional file 2 is shown. Approximately 3 × 10^5^ (APP and medium) and 5 × 10^4^ (PMA/Ionomycin) cells are shown in the contour plots. [file 13567_2017_411_MOESM6_ESM.pdf]

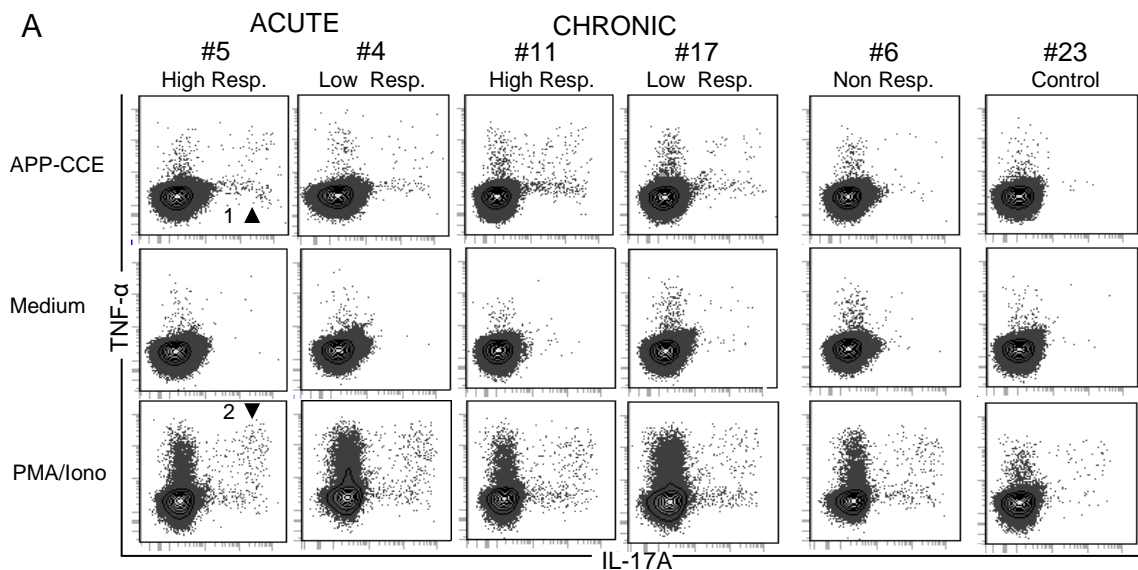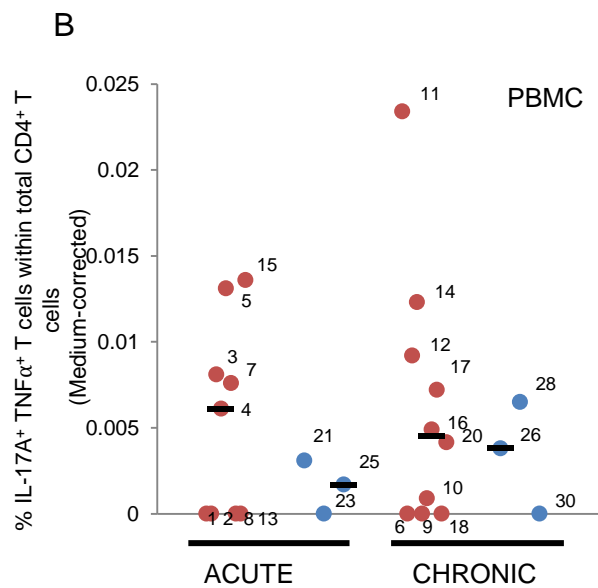

Supplement: Supplementary file 7 — Additional file 7. Co-production of TNF-α and IL-17A by CD4 + T cells in peripheral blood. Phenotyping and intracellular cytokine staining were performed on PBMC following overnight in vitro stimulation (APP-CCE, medium, PMA/Ionomycin). (A) Living CD4+ T cells were gated (not shown; see Additional file 1) and further analyzed for production of TNF-α and IL-17A. Data from the same animals as in Additional file 2 are shown. Approximately 3 × 10^5^ (APP and medium) and 5 × 10^4^ (PMA/Ionomycin) cells are shown in the contour plots. (B) Frequency of IL-17A/TNF-α co-producing CD4+ T cells in PBMC of infected animals (red dots) and control animals (blue dots) during acute and chronic phase. Numbers next to colored dots indicate numbers of individual animals. Median percent values are indicated by black bars. Medium-corrected percentage values are presented (% of IL-17A+ TNF-α+ cells within total CD4+ T cells for APP-CCE stimulation minus % of IL-17A+ TNF-α+ cells within total CD4+ T cells for medium incubation). Arrow heads are introduced in the main text. [file 13567_2017_411_MOESM7_ESM.pdf]

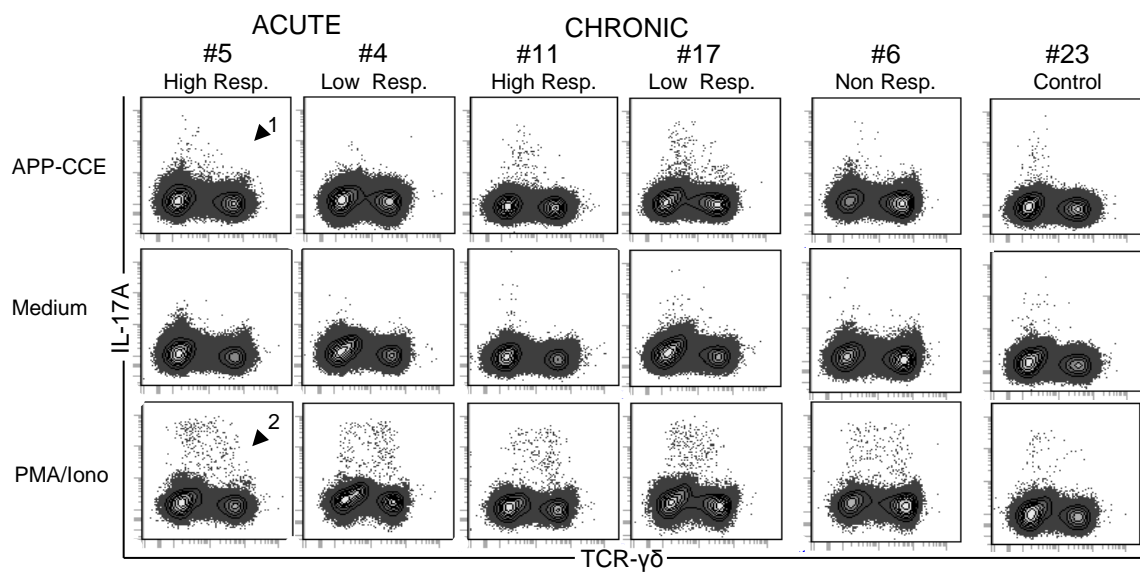

Supplement: Supplementary file 8 — Additional file 8. Production of IL-17A by non-CD4 + cells and γδ T cells in the peripheral blood. PBMC were incubated overnight with APP-CCE, medium or PMA/Ionomycin and subsequently analyzed for CD4, TCR-γδ and IL-17A expression by FCM. Living lymphocytes excluding CD4+ T cells (not shown; see Additional file 1) were gated and further analyzed for expression of IL-17A and TCR-γδ. Data from the same animals as in Additional file 2 are shown. Approximately 7 × 10^5^ (APP and medium) and 2 × 10^5^ (PMA/Ionomycin) cells are shown in the contour plots. Arrow heads are introduced in the main text. [file 13567_2017_411_MOESM8_ESM.pdf]

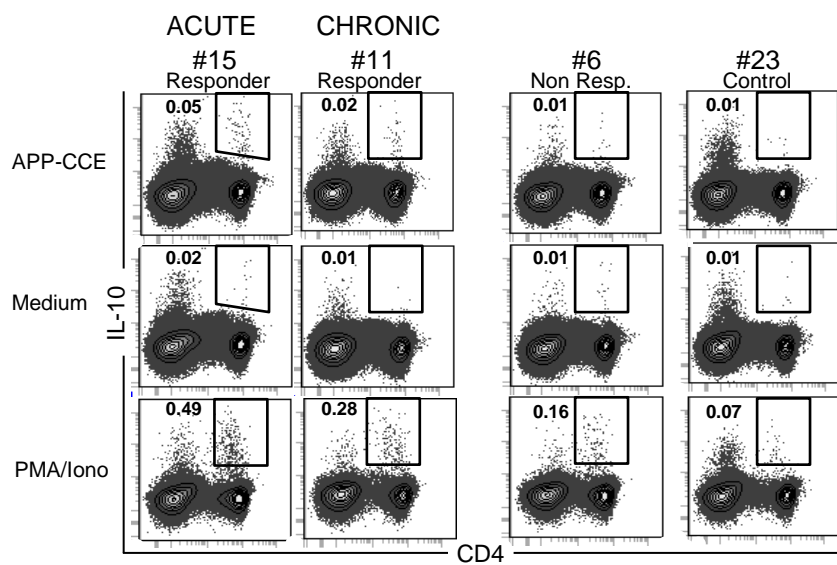

Supplement: Supplementary file 9 — Additional file 9. APP-CCE-specific IL-10-producing CD4 + T cells in peripheral blood. PBMC were incubated overnight with APP-CCE, medium or PMA/Ionomycin and subsequently analyzed by FCM. Living cells were gated (not shown; see Additional file 1) and further analyzed for expression of IL-10 and CD4. Data from representative animals from different groups are displayed: #15 for the acute phase and #11 for the chronic phase, both designated as “responders”; #6, designated as “non-responder” and control pig #23. Approximately 8 × 10^5^ (APP and medium) and 1.5 × 10^5^ (PMA/Ionomycin) cells are shown in the contour plots. Numbers displayed within the contour plots indicate the percentage of IL-10+ CD4+ T cells within total CD4+ T cells. [file 13567_2017_411_MOESM9_ESM.pdf]

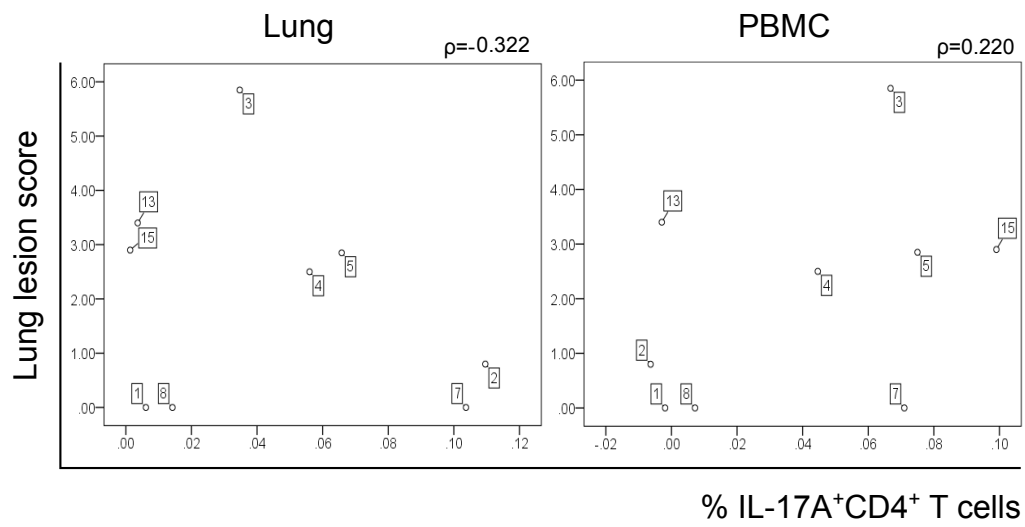

Supplement: Supplementary file 10 — Additional file 10. Correlation of the frequency of IL-17A + CD4 + T cells with lung lesion score during the acute phase. Scatterplots show correlation of the frequency of IL-17A+ CD4+ T cells isolated from lung and blood with lung lesion score in acutely infected animals. Spearman’s Rank Correlation Coefficients (ρ) are displayed above each scatterplot. [file 13567_2017_411_MOESM10_ESM.pdf]

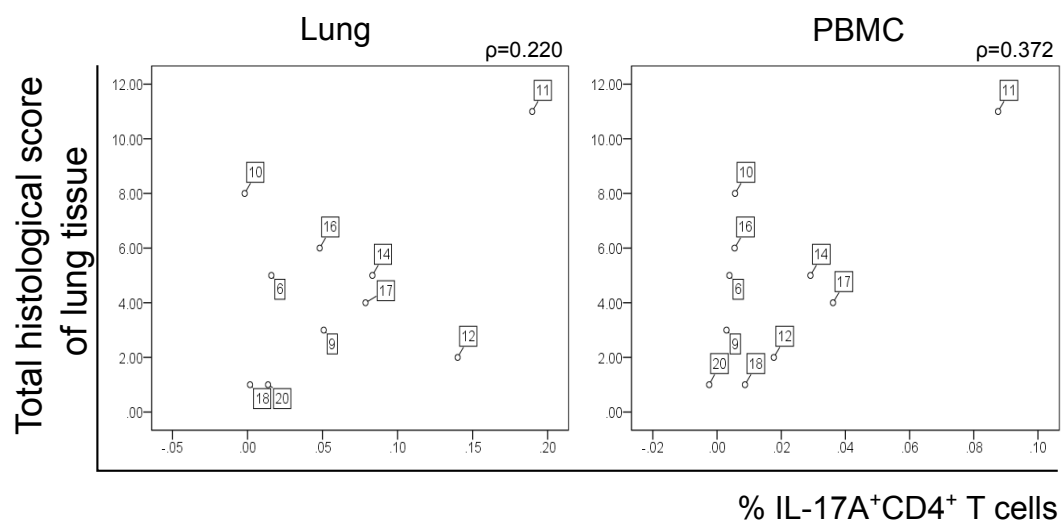

Supplement: Supplementary file 11 — Additional file 11. Correlation of the frequency of IL-17A + CD4 + T cells with histological score of lung tissue during the chronic phase. Scatterplots show correlation of the frequency of IL-17A+ CD4+ T cells isolated from lung and blood during the chronic phase with the histological score of the lung tissue sampled adjacent to tissue used for lymphocyte isolation. Histological scores for each sample were calculated by summing up the grading of all parameters shown in Additional file 4. Spearman’s Rank Correlation Coefficients (ρ) are displayed above each scatterplot. [file 13567_2017_411_MOESM11_ESM.pdf]
