# Supplementary material for: Frequency of Th17 cells correlates with the presence of lung lesions in pigs chronically infected with Actinobacillus pleuropneumoniae
Source: Vet Res. 2017 Feb 6;48:4. doi: 10.1186/s13567-017-0411-z (PMC5294905; doi:10.1186/s13567-017-0411-z)
Supplement: Supplementary file 4 — Additional file 4. Histological evaluation of lung tissue from infected animals. Lung tissue of the dorsal portion of left caudal lung lobe (adjacent to samples used for lymphocyte isolation) was taken from acutely and chronically infected animals. This tissue was paraffin embedded, stained with hematoxylin and eosin, and examined for presence and quantity of parameters A–H (see legend). The quantity and presence of each parameter were assessed by using a score from 0 to 3 (0 = not present, 1 = low grade, 2 = moderate grade, 3 = high grade). No sample in this study presented lesions of grade 3; therefore this grade is not shown in the legend. [file 13567_2017_411_MOESM4_ESM.pdf]

Acute

#	A	B	C	D	E	F	G	H
1	0	0	1	0	0	0	0	0
2	0	0	0	0	0	0	0	0
3	0	0	0	1	0	0	0	0
4	0	1	0	1	1	1	1	0
5	0	0	0	0	0	1	1	0
19	0	2	2	2	2	2	2	0
7	0	1	1	0	0	0	1	0
8	0	0	0	0	0	0	1	0
13	0	1	0	1	0	0	0	0
15	0	2	1	1	1	1	1	0

A Necrosis of lung tissue
B Neutrophilic and histiocytic infiltration of lung parenchyma
C Lymphocellular infiltration of lung parenchyma
D Vascular leakage in alveolar lumen
E Neutrophilic and histiocytic infiltration of lung septa and pleura
F Lymphocellular infiltration of lung septa and pleura
G Vascular leakage in lung septa and pleura
H Fibroplasia

Chronic

#	A	B	C	D	E	F	G	H
6	0	1	1	1	0	1	1	0
9	0	1	0	1	0	1	0	0
10	0	2	2	2	0	1	1	0
11	1	2	2	0	1	2	1	2
12	0	1	1	0	0	0	0	0
14	0	1	1	1	0	1	1	0
16	0	1	1	2	0	1	1	0
17	0	2	1	1	0	0	0	0
18	0	1	0	0	0	0	0	0
20	0	0	0	0	0	1	0	0

Histological scoring of lung tissue

2
1
0

Increasing grade
